# Preparation and optimization of activated nano-carbon production using physical activation by water steam from agricultural wastes

**DOI:** 10.1039/c9ra07409k

**Published:** 2020-01-08

**Authors:** Mohammad Amin Nazem, Masoud Habibi Zare, Saeed Shirazian

**Affiliations:** Department of Chemical Engineering, College of Engineering, University of Isfahan 81746-73441 Isfahan Iran; Department of Chemical Engineering, Isfahan University of Technology Isfahan 84156-83111 Iran; Department for Management of Science and Technology Development, Ton Duc Thang University Ho Chi Minh City Vietnam saeed.shirazian@tdtu.edu.vn; Faculty of Applied Sciences, Ton Duc Thang University Ho Chi Minh City Vietnam

## Abstract

Production of activated nano-carbon from agricultural wastes was studied in this work. To obtain the optimum production conditions by a physical activation method, influence of temperature (850, 900, 950 and 1000 °C), activation residence time (30, 60 and 90 min), and mill rotation (200, 300 and 400 rpm) were investigated using three different raw materials including walnut, almond and pistachio shells. To prepare activated nano-carbon, all the samples were heated up to the final activation temperature under a continuous steam flow of 130 cm^3^ min^−1^, and at a heating rate of 3 °C min^−1^, and were held at the different activation temperatures for 30, 60 and 90 minutes. BET surface area of the obtained activated carbons was measured from nitrogen adsorption data in the relative pressure range between 0 to 1. Activated nano-carbon standard indexes were evaluated according to the ASTM standard and the samples were compared. First, the cellulose raw material was heated in the carbonization furnace at 600 °C and then activated in the advanced activation furnace at a temperature between 850 to 1000 °C for 30, 60 and 90 minutes with water vapor. Ash percentage, iodine content, moisture content, specific area, elemental analysis, and FESEM were used for product characterization. The results of the analysis showed that by using the water vapor physical activation method and optimizing the parameters of this process including time and rotation of the mill up to 10 min and 400 rpm, resulted in a significant increase in specific surface area, cavity volume and the iodine number of the final product.

## Abstract 2

Production of activated nano-carbon from agricultural wastes was studied in this work.

## Graphical abstract

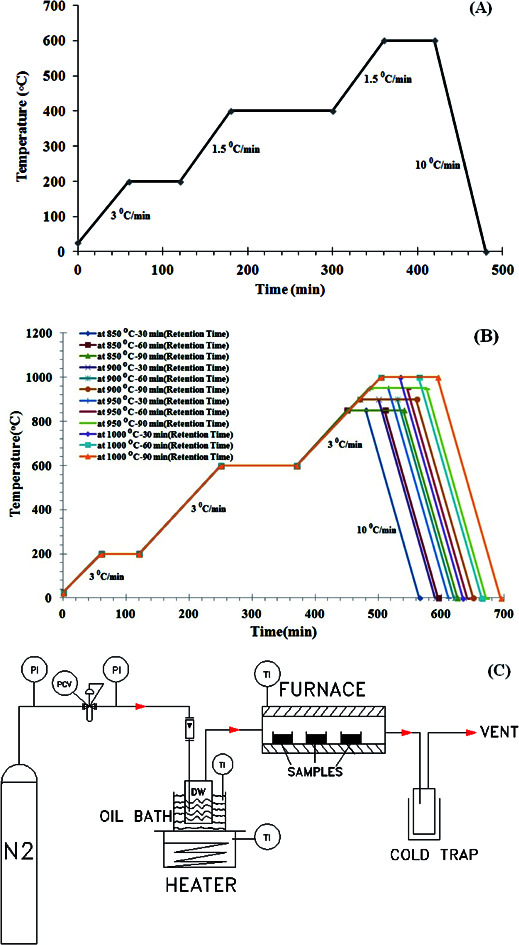

## 1 Introduction

Nowadays, biomass is one of the greatest sources of renewable energy. These important sources including methane (marsh gas), polyethylene glycol, and agricultural waste have been studied in many research works.^1–4^ Porous activated carbon materials are characterized by their high surface area and porosity. Such properties of these materials make the activated carbon useful as an efficient adsorbent for separation/purification applications.^5–7^ Raw materials to convert to activated carbon must be activated after removal of volatiles. Chemical activation is accomplished by impregnating raw materials with acidifying and base agents or mineral salts under low temperature operating conditions.^8^

Biomass (*e.g.* walnut, almond and pistachio shells) is a crop waste generally wasted or incinerated, which results in production of large amount of waste and pollution. A number of scholars have undertaken research into the potential uses of biomass including walnut, almond and pistachio shells to produce activated carbon.^9^ Martínez *et al.* produced activated carbon from walnut shells as biomass.^10^ Production and characterization of high specific surface area activated carbon from coconut shells *via* microwave heating was studied by Yang *et al.*^11^ Okutucu *et al.* produced fungicidal oil and activated carbon from pistachio shells.^12^

Al-Qodah and Shawabkah studied preparation and characterization of granular activated carbon from sludge.^13^ Sulfuric acid was used as chemical activation agent, and the effect of process parameters on the obtained activated carbon was investigated. The obtained activated carbon was highly porous with specific surface area of 580 m^2^ g^−1^. In other works, Yahya *et al.*^14,15^ studied production of activated carbon from desiccated coconut residue using KOH and NaOH as chemical activating agents.

Tyagi *et al.*^16,17^ studied preparation of activated carbon from biowaste as electrocatalyst for oxygen reduction. Sharma and Kar^18,19^ studied oxygen reduction activity of biomass-derived carbon. They used poultry featherfiber and polyvinylpyrrolidone (PVP) as the main source of carbon, and investigated the effect of temperature.^20,21^

Activated carbon, as an adsorbent with a high absorption capacity and low cost, has found many applications in adsorption from liquid phase and/or gas phases. One of the applications of this substance in adsorption from a liquid phase is decolorization of sugar solution, drinking water treatment, wastewater treatment, gas absorption in gas masks, and solvent recovery systems. Various resources can be used as raw material for the production of this product, including cellulosic raw materials such as wood, coconut, core of fruits and other agricultural waste, carbon raw materials such as coal, oil coke, coal bitumen and polymeric raw materials including waste from various types of rubber and plastics can be mentioned.^22–24^ The use of cellulosic raw materials to absorb the liquid phase, mainly when the end product is intended for use in the food industry is less suitable due to its impurities. Activated carbon production is possible using both physical activation and chemical activation methods. The purpose of activation is to create a high free porous carbon structure in the raw material.^25^ In the chemical activation method, which is a single-step method for producing activated carbon, the raw material is mixed with a concentrated solution of an activating agent and the resulting mixture after drying, under the inert atmosphere condition, is heated in an atmospheric furnace. The most important substances used as activating agents include alkali or alkaline earth metal compounds such as potassium hydroxide, potassium carbonate, sodium carbonate, magnesium chloride, and some acids such as phosphoric acid, sulfuric acid, aluminum chloride, and zinc chloride. The role of activating agent here is to remove water from the structure of the primary material and lower the temperature necessary for carbonization, which contributes to the creation of a porous structure in the product.^26^ In this method, the characteristics of raw material such as the type and size of its seeds, the type of activator, the ratio of mixing raw material with the activator (percentage of inoculation), the conditions of drying and heating in the furnace, will have a significant effect in properties of the finished product.^27^ Obviously, the selection of the activation method also affects the porosity of the product. Basically, physical activation method often creates cone-shaped holes, whereas the chemical activation method leads to the formation of cavities in the form of bottles.^22^

This paper presents the conditions of nano-products obtained from the physical activation of raw materials including walnut, almond and pistachio shells using water vapor. Influence of operating conditions such as activation temperature, residence time and high milling speed on the porosity, grain size and adsorption properties of the nano products prepared from all three different walnut, almond, and pistachio crops have been optimally studied. These variables have been identified as highly effective agents in the production of activated nano-carbon. The choice of the high-energy mill as a nano-producing agent has been the reason that this method has already been used in the production of metallic nanoparticles.^28^

## 2 Materials and methods

### 2.1 Materials

The raw materials used in this study were walnut, almond and pistachio shells in Iran with the specifications given in Table 1. In the first stage to make activated carbon, 300 g of Iranian pistachio, almond and walnut shells were prepared.

**Table 1:** Elemental analysis of walnut, pistachio and almond shells samples used as raw materials

	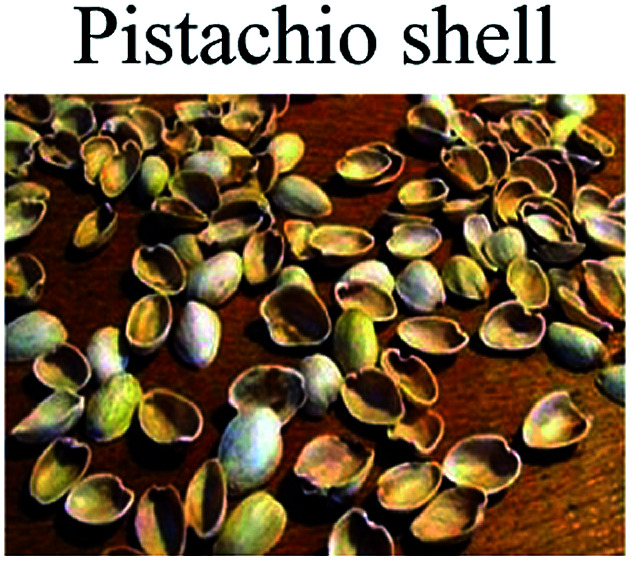	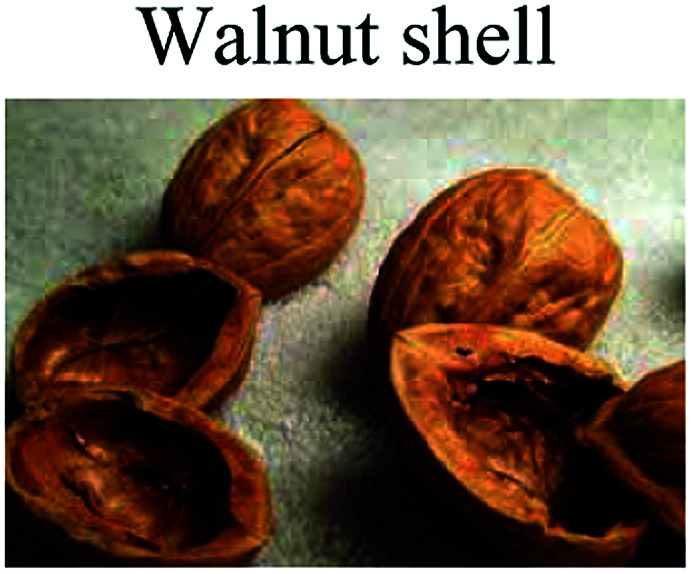	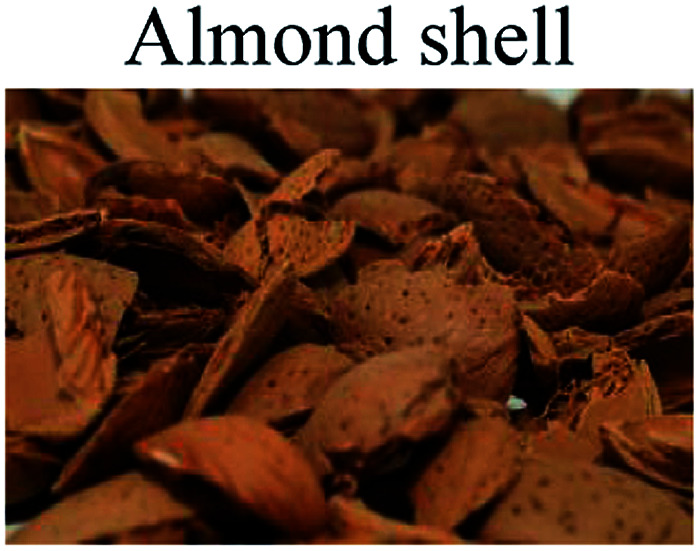
Carbon (%)	51.4	49	50.3
Hydrogen (%)	5.95	5.75	6.05
Cellulose (%)	43.2	36.4	38.8
Hemicellulose (%)	25.5	27.9	29.1
Lignin (%)	16.5	43.7	29.5
Ash (%)^a^	0.49	0.44	0.31
Moisture (%)^a^	8	11	8.7
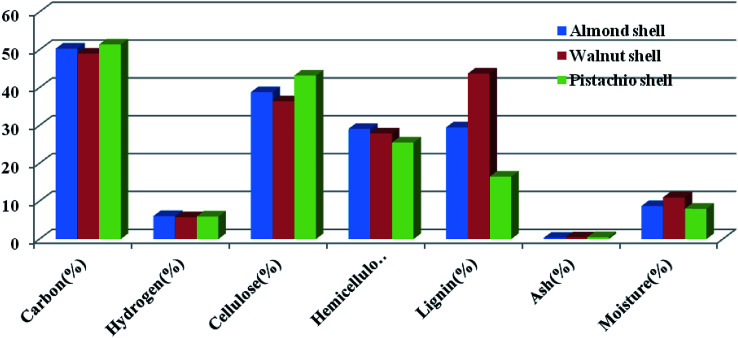			

a Determination of ash content according to standard guidelines ASTM D-2866. Determination of moisture according to standard guidelines ASTM D-2867.

The high carbon content and lower ash content of these three materials indicate their high potential for producing a high-quality carbon. Elemental analysis of the samples in Table 1 shows that the mechanical properties of activated carbon obtained from almond shells may be greater than walnut and pistachio shells. This method does not use chemical materials, which reduces the cost of production, and is a green route to production of activated nano-carbon.

### 2.2 Experiments

**Fig. 1:**
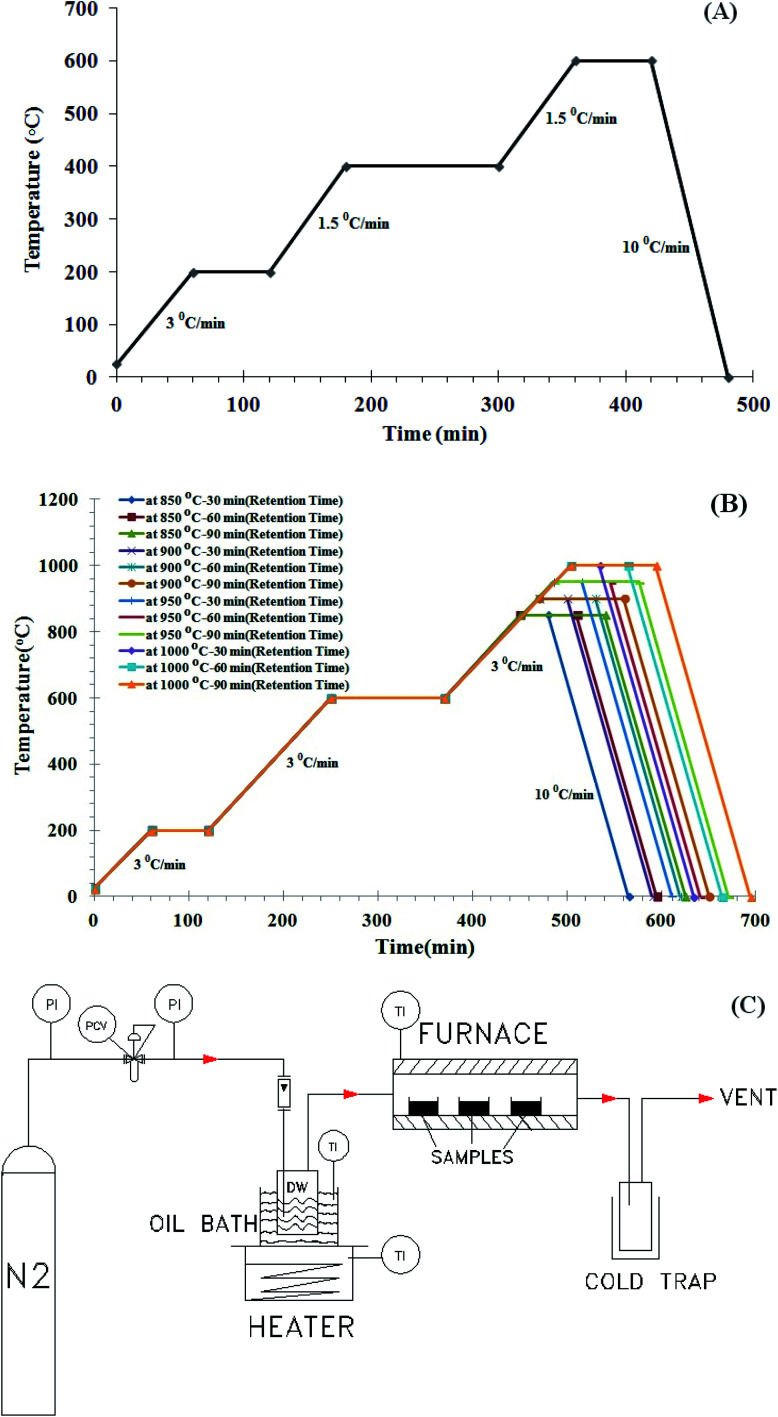
(A) Temperature–time curves for carbonization of walnut, almond and pistachio shells. (B) Temperature–time curves for activation of walnut, almond and pistachio shells. (C) Activated carbon production pilot including atmospheric furnace and steam generating system.

Firstly, to remove impurities and dust, the raw material was washed with deionized water and then dried in an oven at 80 °C. Subsequently, the raw materials crushed by a vibrating mill for 30 minutes, and they are classified by a mesh 20 as regular granules. The prepared granules are washed twice with deionized water and then placed inside the oven at temperature 70 °C for 6 h to completely dry. At this stage, 200 g of each sample was placed in special crucibles, and crucibles was placed into the atmospheric furnace (EXCITION Co., Iran) in order to carry out the process of carbonization (pyrolysis). Then, in order to carry out the carbonization process, the furnace condition was adjusted under nitrogen atmosphere with the specified temperature cycles as shown in Fig. 1A. The samples should be heated up to 600 °C and held at this temperature for 1 hour. Finally, the samples cooled down to ambient temperature with a temperature decay rate of 10 °C min^−1^. In all of the above operations, pure nitrogen gas supplied at the rate of 150 cm^3^ min^−1^ in the furnace for inert atmospheric condition. The products of the carbonization stage have low adsorption capacity due to low temperature carbonization and the presence of bitumen in the pores between the crystals and the surfaces. In the carbonization (or pyrolysis) process, non-carbon components such as hydrogen, oxygen, and volatiles are released from raw materials in the form of gas, and free carbon forms regular groups of graphite crystals. The carbon pore structure is formed at a temperature of about 600 °C. Most of these pores are blocked by the released bitumen during the pyrolysis process. For this reason, the activation stage must be performed in order to open these pores and be used as an adsorbent.

Generally, in the process of carbonization of gases and vapors directly from the carbonaceous structure of the raw material, these gases may react with themselves depending on the temperature and residence time of the carbonization process, which causes secondary products. As temperatures rises, secondary reactions tend to form compounds such as methane, hydrogen, water, and carbon dioxide. If carbonization is carried out at high thermal rates the secondary reactions are so fast that the soot precipitates on the coal particles and coal formed by a combination of a short graphite crystals is irregular. Between the crystals there are small voids that are often not accessible from the outer surface because the cavity network is closed by soot deposition. Therefore, a high heating speed results in a very rapid decomposition, resulting in the production of a solid with a developed network of medium and large pores with low density and abrasion.

The coal produced in the previous stage, after cooling and reaching ambient temperature, was prepared for the activation step. In this step, the conditions for the production were considered at temperature of 850, 900, 950 and 1000 °C, the temperature rise rate was 3 °C min^−1^ and the steam flow rate was 130 cm^3^ min^−1^. Due to the importance of the residence time, three different times were tested according to the corresponding temperatures (shown in Fig. 1B).

The activation process increases the diameter of the cavities created during carbonization, in addition they will be created during the activation of tiny holes, which eventually results in the formation of a cavity structure with a high internal surface area per unit mass of adsorbent. The activation step is accompanied by the removal of inorganic materials and opening of aromatic structure in the early stages of activation and development of the micro-porous structure at the end of activation, so the activation time is very important to complete this process. In the activation process of carbonized materials at temperatures of 850 to 1000 °C with the presence of oxidizing agents of water vapor, the following reactions are involved:

1 C + H_2_O → CO + H_2_

2 CO + H_2_O → CO_2_ + H_2_

These reactions, by consuming some of the available carbon and also removing the residual bitumen from the pyrolysis step, open, expand, and internally bond the carbon pores, thereby significantly increases the internal pore area.

As shown in Fig. 1C, the activated carbon production system includes a nitrogen cylinder, heater and a special container for steam generation, an oil bath, an atmospheric furnace, a crucible and a cold trap to condense exhaust vapors from the furnace. The coal production system is similar to the activation system, with the only difference being that the heater and the oil bath do not have and the nitrogen gas from the cylinder.

In each test, 120 grams of coal produced from each raw material is placed inside the crucible. The codes assigned to P pistachios, samples from walnut, W and samples from almonds are named A. The conditions for each test are shown in Table 2.

**Table 2:** Experimental conditions of activation process of walnut, almond and pistachio shells

Test no.	1	2	3	4	5	6	7	8	9	10	11	12
Activation temperature (°C)	1000			950			900			850		
Furnace atmosphere: water vapor flow (cm^3^ min^−1^)	130											
Temperature rise rate in the furnace (°C min^−1^)	3											
Residence time at activation temperature (min)	30	60	90	30	60	90	30	60	90	30	60	90
Sample code	P1	P2	P3	P4	P5	P6	P7	P8	P9	P10	P11	P12
	W1	W2	W3	W4	W5	W6	W7	W8	W9	W10	W11	W12
	A1	A2	A3	A4	A5	A6	A7	A8	A9	A10	A11	A12

After completion of the activation and cooling the samples, the samples were washed with very dilute hydrochloric acid (0.01 molar) and deionized water (DW) and dried completely in oven at 70 °C for 2 hours. In this step, activated carbon obtained, according to the specified codes, was placed inside the vibrating milling chamber (at a speed of 400 rpm) and polymer balls of different sizes. The weight ratio of the ball to powder was 10 to 1. Each of the samples was milled for 12 hours. In order to determine the specific surface area by BET, activated carbon samples were subjected to nitrogen adsorption experiments at 77 K. This experiment was carried out by ASAP 2000 equipment (Micrometrics Co.). Determination of Iodine Number has been performed according to ASTM-D4607 standard. Also, for more accurate examination of the structure of the obtained products and raw material samples, the images were taken by the scanning electron microscope (SEM-360), Cambridge, and electron microscopy FESEM (XL30) manufactured by PHILIPS Co. In this case, the particle size was micro-level. Therefore, all of the samples were tested on a specific surface area to select the best samples for the milling process in the planetary ball mill. Samples with the highest specific surface area and the most porous and finer particles were selected for milling in high-energy planetary mills. 50 g of each sample were placed separately in the planetary ball mill. The weight ratio of ball to powder was also 10 to 1, and steel balls of different sizes (5 to 20 mm) were used. In order to test and investigate the effect of parameter of mill speed and therefore the energy applied to the powders, three rounds of 400, 300 and 200 rpm were selected for testing. The optimum time for the ball mill was also selected for 10 minutes. Finally, in order to evaluate the results of experiments, the tests and analyses previously mentioned were performed on the samples obtained.

## 3 Results and discussion

### 3.1 Specific surface area test results of samples (BET) after vibration milling

**Fig. 2:**
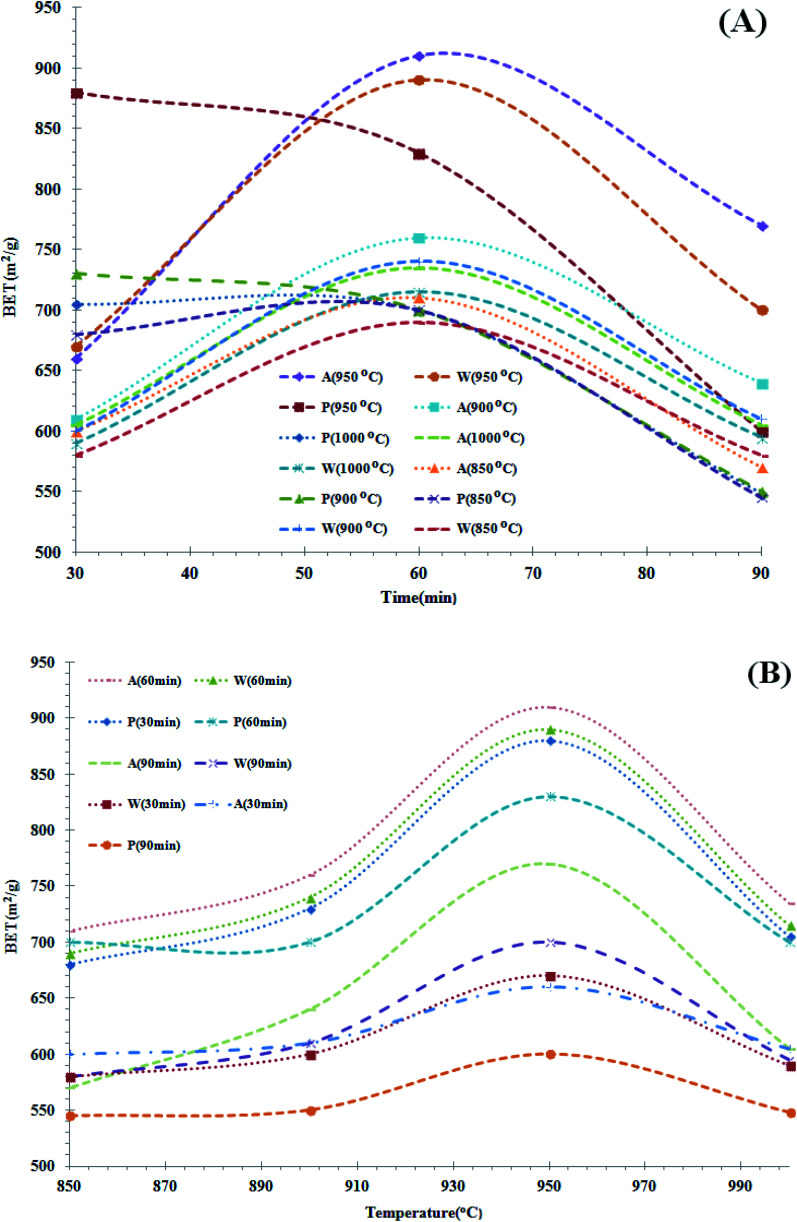
(A) Specific surface area test results and residence time changes (after vibration milling). (B) Specific surface area test results and temperature changes (after vibration milling).

The specific surface area test results of the activated carbon samples obtained from the walnut, almond and pistachio shells after exposure to vibrating mills are shown in Fig. 2. According to Fig. 2A, the results show that during the 30 min residence time (at 1000 °C), the highest specific area was of activated carbon produced from pistachio shell and then almond and walnut shells. The highest specific surface area at 60 and 90 min residence time belong to activated carbon produced from the almond shell and the carbohydrates of walnut shell origin are in the second rank. Except for pistachio shell samples, by increasing the residence time from 30 min to 90 min, specific area of walnut and almond samples increased and then severely declined. The reason for the sharp decrease in the specific surface area of the specimens is that increasing the residence time of the furnace causes the formation of a molten material (intermediate material), which will cover some pores of the activated carbon surface, thereby reducing its specific surface area. The optimum conditions for activation of the pistachio shell (at 950 °C) are 30 minutes, and for the almond and walnut shells are 60 minutes. The most important reason for the optimum condition difference between walnut, almond and pistachio shell samples is due to shell structure and its composition. According to Fig. 2 (at 950 and 900 °C), the results show that decreasing the furnace temperature has an adverse effect on the specific surface area of the specimens and drastically reduces the specific surface area of the specimens. As shown in these graphs, decreasing the furnace temperature reduced the effect of activation time and decreased the slope of the graphs. According to the specific surface area of commercial activated carbon (400 to 1300 m^2^ g^−1^), the results of specimen surface area are favorable and the results of electron microscopy confirm this claim. According to Fig. 2B, the best results are the specific surface area of activated carbon samples at 950 °C activation temperature which is respectively for almond, walnut (60 min in residence time), and pistachio shells (30 min in residence time). According to the above results, the highest specific surface area was obtained at an optimum temperature of 950 °C and an optimum residence time of 30 minutes for pistachio shell and 60 minutes for walnut and almond shells.

The specific surface area decreased for all 4 pistachio shell samples at 850 to 1000 °C with increasing retention time. Increasing the activation temperature and increasing the retention time is not a desirable factor to increase the specific surface area of the pistachio shell and the optimum temperature and time for the pistachio shell is 950 °C and 30 minutes. The specific surface area for all 4 almond shell samples at temperatures of 850 to 1000 °C has an increase-decrease slope with increasing retention time, with the optimum amount being at 60 minutes. Increasing the activation temperature and increasing the retention time is not a desirable factor to increase the specific surface area of the almond shell and the optimum temperature and time for the almond shell is 950 °C and 60 minutes. The special surface behavior obtained from the walnut shell is exactly the same as the almond shell. Given the different structures of the almond shell compared to walnut and pistachio shell samples and that the almond shell has a high pore structure and low density, it can be concluded that this porous structure will have a higher specific surface area than the dense walnut and pistachio shell structure. Given the amount of hemicellulose in the almond shell and the results in the figure, it can be concluded that the thermal stability of almond shell is higher than walnut and pistachio, respectively.

Therefore, the samples obtained at 950 °C represent finer and more porous powders, so we chose these samples to continue the experiment in high-energy milling in a planetary mill.

Overall, it can be concluded that after the carbonization process, completely for all three samples of almond, walnut and pistachio shell (with moderate heat rise rate), performing the activation process at different temperatures and retention times shows different behavior in terms of the specific surface area of each sample. However, in general the increase in temperature and the increase in retention time cannot increase the specific surface area of the specimens and it depends on the type of raw material. Each sample according to the type of structure (texture, hardness, brittle, dense and spongy), lignin, hemicellulose and cellulose and under different process conditions it reaches the most optimal product with the highest specification. The results show, given the similar structure of almond and walnut shells, these two samples exhibited the same behavior *vis-à-vis* the retention time of the activation process (the optimal time being 60 min). Different behavior of pistachio shell and the increase in retention time had an adverse effect on the specific surface area of pistachio shell samples (Fig. 2A). All three samples of almond, walnut and pistachio shells exhibited similar behavior against increasing temperature of activation process and reached their highest specific surface temperature at 950 °C (Fig. 2B). Also, the vibrating milling process after fully carbonization and activation, by applying energy and due to the fragile structure of the specimens, it was able to reveal holes and pores that did not have access to the outer surface of the specimen and reduce the particle size to a micron level and increases the specific surface area of the samples but because of insufficient energy, this amount did not reach the desired level.

### 3.2 Burn off percentage

The burn-off parameter indicates the process efficiency and the available weight of activated carbon product relative to the initial weight of the material used for activation. To calculate the burn off percentage, the following formula was used:3
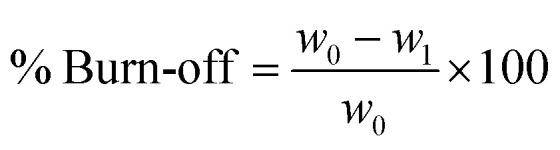
*w*_0_ = coal weight before activation, *w*_1_ = coal weight after activation.

**Fig. 3:**
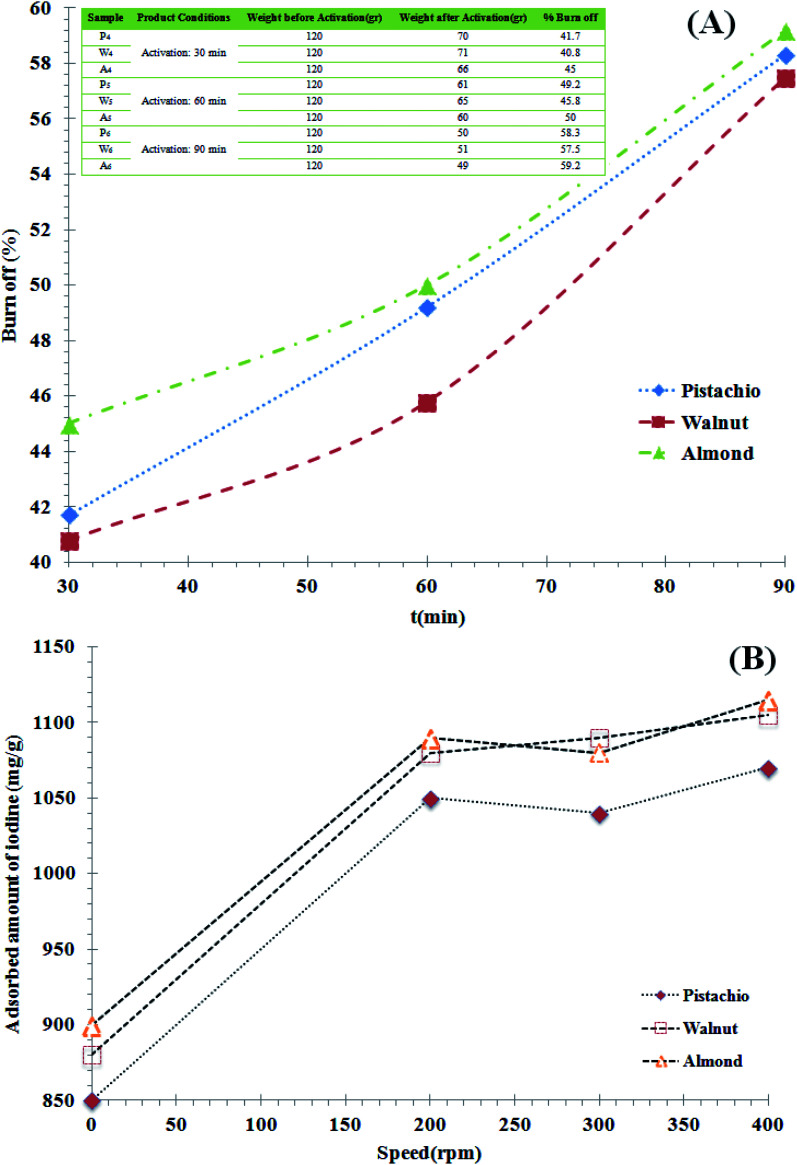
(A) Experimental results and changes in burn off percentage from almond, walnut and pistachio shells. (B) Changes in the iodine number of samples made of activated nano-carbon *vs.* planetary mill speed.

The initial weight of all samples was 120 g for activation. Since the optimum temperature for activation was 950 °C, the samples produced at this temperature were selected for further experiments. For this reason, their weight after activation is shown in Fig. 3A. As expected and the results are clear, with increasing residence time, the percentage of burn-off increases because more volatiles are removed from the coal and burned over time. Therefore, increasing the residence time from one value to another not only reduces the specific surface area, but reduces the efficiency and increases the burn off. The highest percentages in each of the three states were for carbonates with almond bark origin and then for pistachio carbonates. In other words, the production of activated carbon from walnut bark has a lower burn off and higher yield (see Fig. 3A).

### 3.3 Determination of iodine number of activated carbon samples

The iodine number (IN) is measured based on the ASTM D4607-94 approach. The IN is determined as the mass of iodine adsorbed (mg) by one gram of carbon when the iodine concentration of filtrate is 0.02 N. IN approach of measurement is based upon a three-point isotherm, in which standard iodine solutions are prepared and treated with 3 various weights of activated carbon under predetermined conditions. Fig. 3B shows the iodine number variations of the samples made *versus* mill speed. This figure shows a sharp increase in iodine (for all three almond, walnut and pistachio samples) up to 200 rpm, and since then, changes in product iodine numbers have not shown a regular increase. Changes in the dimensions of the raw material particles have not shown a considerable influence on the adsorption properties of the activated carbon. IN is actually considered as a relative measure of activated carbon porosity, and is used to estimate the free surface of some activated carbon samples. For this reason, the trend of iodine number changes is consistent with free surface changes based on BET method. In the first step (by increasing the speed of the mill), the expansion of the small free surface cavities increases the iodine uptake, and in the next step, the iodine number does not change significantly due to the decrease in surface expansion intensity.

### 3.4 Specific surface area test results of samples (BET) after planetary milling

**Fig. 4:**
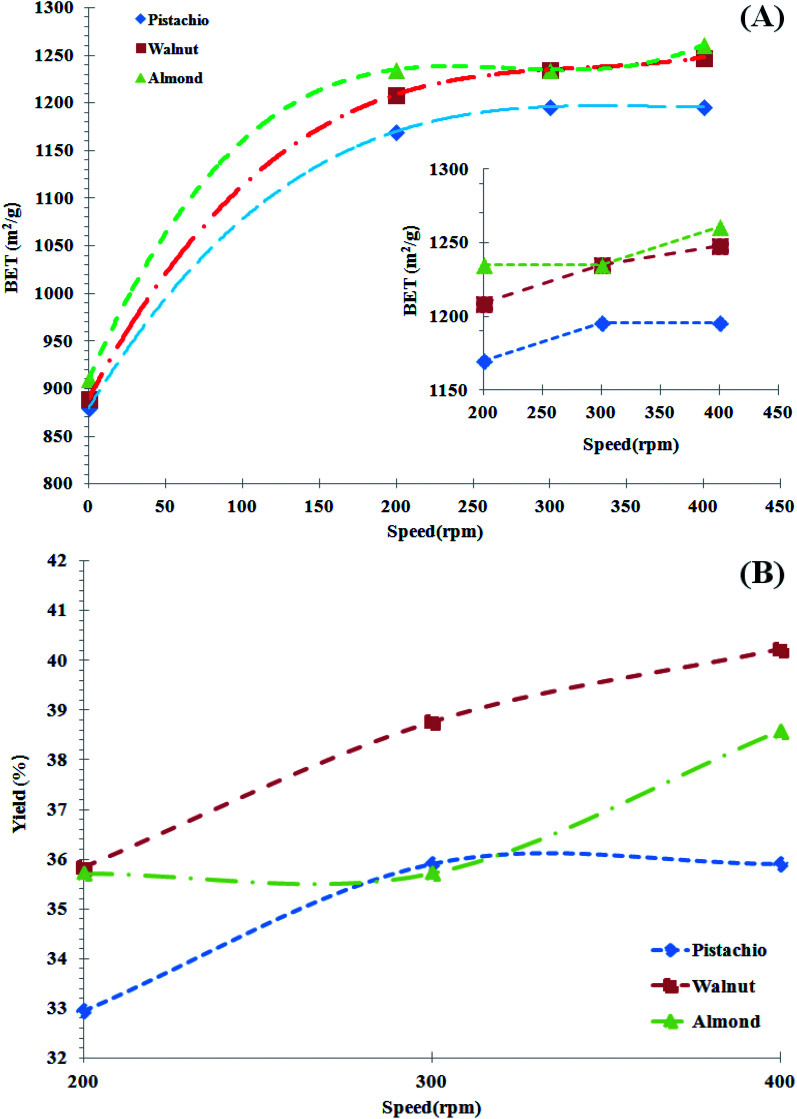
(A) Results and changes of specific surface area testing of samples after milling. (B) Yields results specific surface area changes of samples after milling. Note: in the case of sample coding methods, A, W, and P samples represent almond, walnut and pistachio shells raw material, respectively. The first index relates to the activation conditions at 950 °C, (index 4 represents a 30 minute standby, index 5 stands at 60 and index 6 stands at 90 minutes at the activation stage). The second index represents the conditions around of the planetary mill, (index 1 corresponds to speed 200 rpm, index 2 corresponds to speed 300 rpm and index 3 represents 400 rpm).

Given the greater importance of the two parameters of mill rotation and residence time, in the first step, all parameters are considered constant and only the mill rotation effect is evaluated. The residence time of 10 minutes, the weight ratio of the balls to powder 10 to 1, the steel balls of different sizes, were considered as fixed process parameters and the samples were tested in 3 different speeds. The results of the specific surface and its yields for the samples obtained from the planetary mill are shown in Fig. 4. The BET surface areas of the active nano-carbons produced from biomass raw materials were found to be between 1170 and 1261 m^2^ g^−1^. The highest surface area was determined with a round ratio of 400 rpm, at the activation temperature of 950 °C (W53). As shown in Fig. 4A, the BET increased for all three activated samples obtained from almond, walnut and pistachio shell after milling. As shown in this figure, for all three samples, BET increased slightly with increasing mill speed and 300 rpm was the best condition for active nano-carbon production. Comparing the results of Fig. 2 with Fig. 4 indicates that the particle specific surface area increased. In other words, the use of planetary mills has caused the particles to smaller size. The highest increase in the specific surface area at the high-energy milling stage for almond and walnut shells samples was 38% and 40%, respectively (shown in Fig. 4B).

In other words, the activated carbon produced by these two materials was fragile. As the milling speed increases, smaller particles with a higher specific surface are produced. In the case of pistachio shell, increasing the round from 300 to 400 rpm did not have a significant effect on specific surface. In the case of walnut shell, the increase in speed of mill has consistently increased the specific surface area. In the case of almond shell, an increase from 200 to 300 rpm had no little effect on increasing the specific surface, but an increase from 300 to 400 rpm led to an increase. The different effect of specific surface increase is due to the structural and physical differences of the raw materials. Although with increasing speed, the specific surface of all samples has increased, this increase is not very noticeable and has not increased dramatically. This may have been due to crushing of the particles and subsequently some of the cavities during the grinding process. Increasing the round from 300 to 400 at 10 min residence time did not have the ability to the finer particles, and with an increase in residence time, particles aggregate to form larger sizes and adopt a new spectral distribution. Increasing the round does not have much effect on increasing the specific surface area, so if low-rounds are also the desired size, the use of low-rounds would make more sense because of lower power consumption.

### 3.5 Elemental analysis

According to different process conditions, three samples of P41, W52 and A53 were selected for elemental analysis tests. The test results are listed in Table 3.

**Table 3:** Elemental analysis results of P41, W52, A53 samples

	Elt	Line	Int	Error	*K*	W%	A%	Pk/Bg
P41	C	Ka	445.6	174.5755	0.9411	83.59	87.90	146.75
	O	Ka	33.6	180.3466	0.0348	14.37	11.34	26.13
	Si	Ka	20.4	0.5477	0.0073	0.61	0.28	4.56
	P	Ka	16.3	0.5557	0.0066	0.56	0.23	4.20
	Ca	Ka	10.7	0.4851	0.0074	0.60	0.19	4.34
	Fe	Ka	1.7	0.3070	0.0028	0.27	0.06	2.55
					1.0000	100.00	100.00	
W52	C	Ka	182.3	51.4484	0.5436	57.54	68.01	225.86
	O	Ka	110.0	53.1491	0.1607	27.38	24.30	55.09
	Al	Ka	547.0	1.5647	0.2633	13.48	7.09	49.07
	Si	Ka	19.7	1.5879	0.0099	0.51	0.26	4.38
	K	Ka	5.8	0.2610	0.0051	0.23	0.08	2.74
	Ca	Ka	7.8	0.2646	0.0076	0.34	0.12	3.22
	Fe	Ka	4.1	0.3675	0.0099	0.50	0.13	3.02
					1.0000	100.00	100.00	
A53	C	Ka	144.9	55.2656	0.5046	55.56	66.63	200.18
	O	Ka	97.8	57.0926	0.1669	27.69	24.93	57.03
	Al	Ka	492.2	2.0683	0.2766	14.19	7.57	47.01
	Si	Ka	17.6	2.0990	0.0104	0.54	0.28	5.24
	K	Ka	5.9	0.4341	0.0060	0.28	0.10	2.71
	Ca	Ka	8.0	0.4400	0.0091	0.41	0.15	2.2
	Fe	Ka	9.5	0.3205	0.0264	1.33	0.34	3.1
					1.0000	100.00	100.00	

The above results show that carbon and then oxygen have the highest percentage. The results of Table 3 also show the aluminum impurity, which is related to the impact and the addition of metal impurities due to the friction of the pellets and the grinding chamber. Because the P41 sample was the first to be placed in the mill, no impurities were observed.

### 3.6 Morphology analysis using SEM images

#### 3.6.1 SEM of specific surface area (BET) after vibration milling

**Fig. 5:**
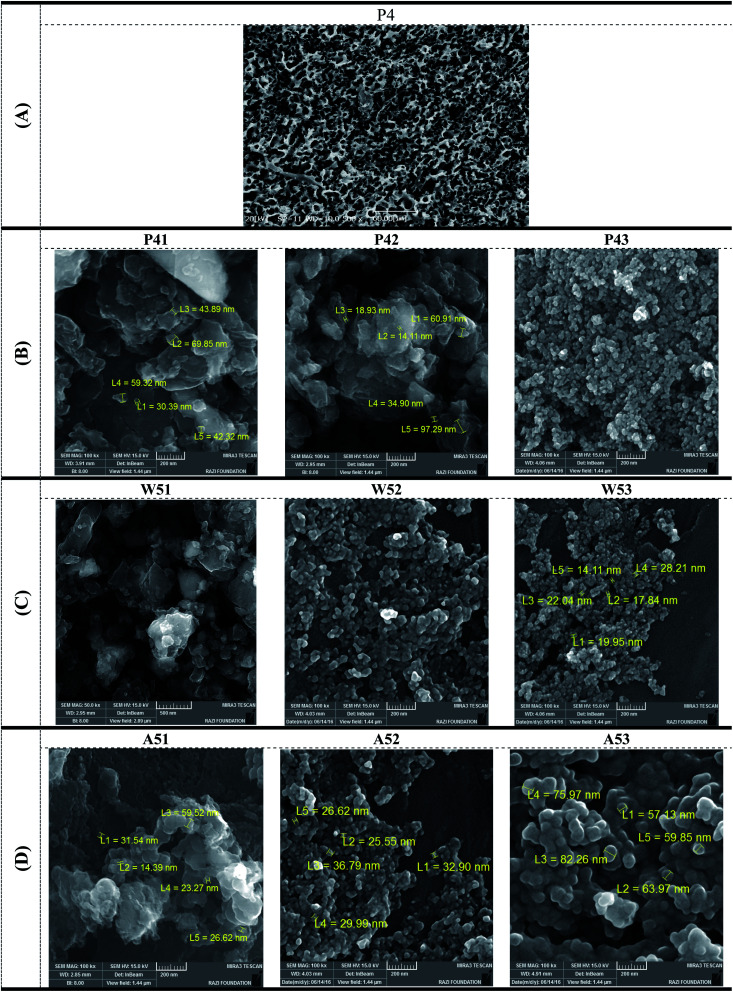
(A) SEM micrographs of a surface of P4 (after vibration milling). (B) FESEM image of crushed activated nano-carbon, P41 and P42 and P43 samples (after planetary ball mill). (C) FESEM image of crushed activated nano-carbon, W51, W52 and W53 samples (after planetary ball mill). (D) FESEM image of crushed activated nano-carbon, W51, W52 and W53 samples (after planetary ball mill).

The SEM image of activated carbon after vibrating mill, P4 sample, is presented in Fig. 5A. The particle sizes for P4 sample is determined 5–20 μm that shows the vibrating mill can not create the activated carbon in range of nanosized. For this reason, the supplementary stage of the planetary mill which is a high-energy mill, was used. This figure clearly shows the porous tissue of this crude material due to its cellular structure. The presence of this porous structure permits the penetration of the activating agent (water vapor) into the internal portion of the raw material, which will ultimately improve the quality of the activation process and thus achieve a high-level product.

#### 3.6.2 Results of electron microscope images after planetary milling

The results of the electron microscope images of the selected samples are shown in Fig. 5. Depending on the dimensions, TEM or FESEM should be used. The morphology of samples was analyzed by scanning electron microscopy (SEM). The FESEM images of activated nano-carbon after planetary ball mill, P41 and P42 and P43 samples, are presented in Fig. 5B, W51, W52 and W53 samples, are presented in Fig. 5C and A51, A52 and A53 samples, are presented in Fig. 5D.

Under the experimental conditions at 200 rpm, the W51 sample had the best results in terms of structure, size, and morphology, and the P41 sample had the worst result. In W51 sample, most nanoscale particles are obtained. In the A51 sample, nanoparticles were produced in good form, but some of them were stuck together and in a bundle state. Under the test conditions at 300 rpm, W52 and A52 samples also had the best results, and with increasing round, smaller particles were produced, while less clustering is observed. The P42 sample, although better than the previous one, has larger particles than the other two samples. Under the test conditions at 400 rpm, the results are largely similar, but the A53 sample has smaller particles (mostly below 50 nm) and has a fewer particles and adhesion. In the other two samples, most particles are between 50 and 100 nanometers. Comparison of the images revealed that the samples from the pistachio shell had larger particles. The above images are in good agreement with the specific surface test results. As the specific surface area of the sample is increased, the particles become smaller. This process is visible in all images. From agricultural waste such as pistachio, almond and walnut shells, activated carbon is produced by water vapor activation method and nano-sized activated carbon is produced by planetary milling (mechanical method).

Based on the results of the activated nano-carbon products, these results can be compared with similar research works, as shown in Table 4.

**Table 4:** Comparisons of results obtained in this work and literature

Raw Material	Particle-size distribution	Carbonization conditions	Activation conditions	Activating agent	Additional information	*S*_BET_ (m^2^ g^−1^)	Reference
Hazelnut shell	Larger than nano-sized	N_2_, 550 °C h^−1^	**—**	ZnCl_2_	Activated carbons after washing with dilute HCl solution and hot distilled water	647	29
Palm kernel shell	Larger than nano-sized	N_2_, 700 °C/30 min	500–900 °C	KOH	Optimal condition of impregnation ratio (1 : 1.5 raw/KOH) and activation temperature of 800 °C	217	30
Durian shell	Larger than nano-sized	**—**	400–500 °C/30 min	H_3_PO_4_	—	1024	31
Bagasse	Larger than nano-sized	N_2_, 500 °C/1 h	900 °C/10 min	ZnCl_2_	—	923	32
Pistachio shell	Larger than nano-sized	N_2_, 250–1000 °C/2 h	900 °C/30 min	CO_2_	One-step pyrolysis/activation	778.1	33
Almond shell	Larger than nano-sized	N_2_, 600 °C/1 h	850 °C/30 min	Steam	Chars were subsequently into contact with diluted N_2_ + H_2_O, steam partial pressure equal to 0.61	601	34
Walnut shell	Larger than nano-sized	N_2_, 600 °C/1 h	850 °C/30 min	Steam	Chars were subsequently into contact with diluted N_2_ + H_2_O, steam partial pressure equal to 0.61	792	34
Pistachio shell	50–100 nm	N_2_, 600 °C/1 h	950 °C/30 min	Steam	After milling in a planetary mill under 400 rpm for 10 minutes	1196	Present work
Almond shell	50–100 nm	N_2_, 600 °C/1 h	950 °C/60 min	Steam	After milling in a planetary mill under 400 rpm for 10 minutes	1261	Present work
Walnut shell	50–100 nm	N_2_, 600 °C/1 h	950 °C/60 min	Steam	After milling in a planetary mill under 400 rpm for 10 minutes	1248	Present work

As can be seen from the results of Table 4, the obtained activated carbon in this study is very suitable for the specific surface area and size of the nanoparticle compared to the activated carbon obtained in similar work based on physical and chemical activation processes. Also the used process in this study is low cost, and due to non use of chemical materials is environmentally friendly. One of the major and expanding applications of activated carbon and especially nano-sized activated carbon is in the use of supercapacitors. For this reason, the obtained activated nano-carbon will have the potential to be used in supercapacitors.^35^

## 4 Conclusions

Using physical activation with water vapor and raw materials used in this study, activated carbon granules with high specific surface area was obtained. The highest specific surface area of activated carbon were obtained for pistachio shell during the 30 min activation time and 60 min for almond and walnut shell. The highest specific surface area of activated carbon was obtained after activation and before high-energy milling, 910 m^2^ g^−1^, which belongs to the almond shell with a 60 min residence time. Walnut shell had the best yield and lowest burn-off and almond shell had the lowest yield and highest burn-off. The vibrating mill has not been able to produce nano activated carbon particles, and finally, with a high residence time up to the micro scale (above 100 nm) it can mill these particles. High energy mills (planetary mills) are capable of producing activated nano-carbon. As the mill round increases, the specific surface area of the powder increases and smaller particles are produced. The highest specific area after planetary milling was for almond and walnut shell, respectively, at 400 rpm (1261 and 1235 m^2^ g^−1^, respectively), but the optimum milling speed was 300 rpm. Adjusting the dimensions of the holes and how they are distributed plays a major role in the process of activated nano-carbon production because these features affect the end-use of the product. Activated carbon with fine holes smaller than 2 nm is suitable for adsorption from the gas phase, whereas a sample with high aggregation of medium holes with dimensions ranging from 2 to 50 nm is more suitable for adsorption in liquid phase. Almond shell as a cellulosic material with a cellular and porous structure has shown a very good potential as a raw material for the production of highly specific surface activated nano-carbons. Changing the type of milling of samples, results in a significant increase in the free surface area and volume of the cavities. The iodine content of the products has also increased significantly. Given the near-surface specificity of the two samples, the particle size of the two samples was predicted to be similar, and the FESEM results confirm this prediction. Finally, using the assumptions and methods adopted in this study, one can achieve nano-sized activated carbon powder by using agricultural wastes such as pistachio, almond and walnut shells, by physical activation with water vapor and high-energy mechanical milling.

## Conflicts of interest

There are no conflicts to declare.

## Supplementary Material
